# Lanthanum delays senescence and improves postharvest quality in cut tulip (*Tulipa gesneriana* L.) flowers

**DOI:** 10.1038/s41598-020-76266-0

**Published:** 2020-11-10

**Authors:** Fernando Carlos Gómez-Merino, Ana María Castillo-González, Maribel Ramírez-Martínez, Libia Iris Trejo-Téllez

**Affiliations:** 1 Laboratory of Plant Nutrition, College of Postgraduates in Agricultural Sciences Campus Montecillo, 56230 Texcoco, Mexico; 2 Institute of Horticulture, Chapingo Autonomous University, 56230 Texcoco, Mexico

**Keywords:** Plant sciences, Plant physiology

## Abstract

We tested two sources of lanthanum (La), LaCl_3_ and La(NO_3_)_3_ × 6H_2_O at a concentration of 40 µM each, in the treatment solution of cut flowers of 15 tulip (*Tulipa gesneriana* L.) cultivars. Ascorbic acid (AsA; 0.2 g/L) was used as a reference solution, while distilled water was evaluated as an absolute control. With both La sources, bud length and diameter, and stem length were increased; as a result, stem curvature was also significantly increased with La treatments. The cultivars Laura Fygi and Rosario registered the highest relative stem elongation. Lalibela and Acropolis displayed the greatest stem curvature on the last day in vase. At 3, 5, 7, 9 and 11 days after cutting, the highest solution uptake was recorded in flower stems treated with LaCl_3_, surpassing the control by 5, 11, 15, 18 and 24%, respectively. The relative stem elongations observed were 21.3, 27.4, 35.2 and 35.5% in the control, AsA, LaCl_3_ and La(NO_3_)_3_, respectively. The mean solution uptake per gram of stem fresh biomass weight was 1.44, 1.44, 1.71 and 1.54 mL in the control, AsA, LaCl_3_ and La(NO_3_)_3_, respectively. LaCl_3_ significantly increased the bud length and solution uptake of flower stems, while La(NO_3_)_3_ × 6H_2_O increased stem fresh weight.

## Introduction

A preservative solution is used to control ethylene synthesis and microbial proliferation, maintaining an adequate water and respiratory balance as well as the color of petals, while boosting the antioxidant system and inducing opening of the floral buds^1^. Importantly, management of cut flowers in preservative solutions helps keep the stems for longer periods of time, and thus prolongs the vase life of flowers^2^. These solutions mainly include sugars, acidifiers and germicides, while other compounds may also be added to induce antioxidant responses^3,4^. In order to test the individual effect of a novel compound, treatment solutions have a simpler composition than preservative ones. Once the beneficial effect of such a compound is experimentally demonstrated, it can be incorporated into a prepared preservative or holding/vase solution.

In terms of postharvest handling and preservation of cut flowers, L-ascorbic acid (AsA) has been proved n ot only to act as an important antioxidant, but has also been linked to developmental senescence and programmed cell death through a complex signal transduction network^5^. Consequently, AsA has been used successfully in postharvest management of different cut flowers^5,6^. In cut lisianthus (*Eustoma grandiflorum* [Raf.] Shinn) flowers, stems exposed to 200 mg/L AsA exhibited the maximum solution uptake and the highest dry matter weight^6^, while with 300 mg/L AsA flowers displayed the longest vase life and highest petal water content^7^.

**Figure 1:**
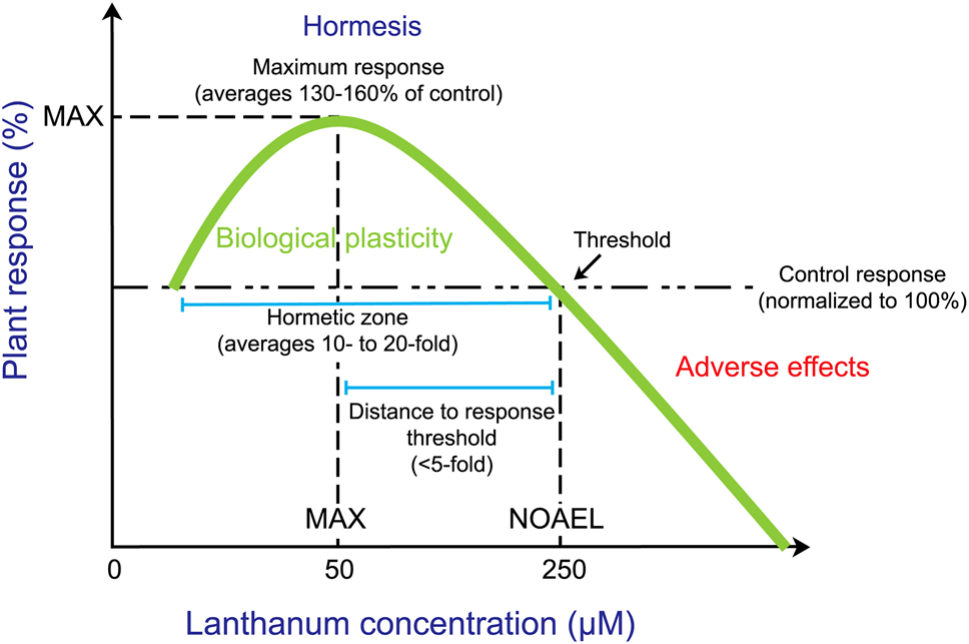
Schematic model of a representative idealized biphasic dose–response hormetic curve for the effect of lanthanum (La) on plants. MAX: Maximum biological response to low La concentrations, which is commonly below 150% of control response. NOAEL: No-observed-adverse-effect-level. The MAX:NOAEL distance is often below fivefold, with a geometric mean of 4.5-fold. The model is based on experimental data previously analyzed^18,20,54^.

Beneficial elements can also retard senescence and preserve cut flowers^8^. Among them, lanthanum (La) can be incorporated into the vase solution during postharvest handling of cut flowers, though its biological functions and effects on tulip (*Tulipa gesneriana* L.) have been little studied^9^. Lanthanum belongs to the Lanthanides (Ln^3+^), which are affiliated to the Rare Earth Elements (REE). The 17 chemical elements that make up the REE group have similar chemical and physical properties^10^. The symbol Ln^3+^ is often used as a generic representation of Lanthanides since they typically have trivalent oxidation states. Generally, the parent materials have REE compositions ranging from 0.1 to 100 mg/kg^11^. Considering the chemical properties of La and the physiological effects it causes in higher plants, La^3+^ may display similar functions to those of Ca^2+^ and K^+^^12^. For instance, in bell pepper (*Capsicum annuum* L.), the application of La increased the number of flowers^13^, which may be correlated with increased endogenous concentrations of cytokinins (important components of floral stimuli in plants)^14^. In Easter lily (*Lolium longiflorum* Thunb.), the use of LaCl_3_ delayed senescence of cut flowers by stimulating their antioxidant defense system and water retaining capacity^15^. Lanthanum may also influence the gravitropic response of cut tulip stems, while stem bending is dependent on the cultivar and is positively correlated with the rate of postharvest stem elongation. Since La reduces stem elongation, LaCl_3_ prevents stem bending^16^. In cut tulip flowers cv. Ile de France, stem diameter and length were increased in comparison to the control, by supplying them with 10 µM La in the nutrient solution^17^. Although no significant differences in stem diameters among treatments were found, plants treated with nutrient solutions containing 10 µM La showed diameters 2% bigger than the control plants. Indeed, La may stimulate plant growth within a biphasic dose–response hormetic curve^18^, which is characterized by a low dose stimulation or beneficial effect and a high dose inhibitory or toxic effect^19^. After analyzing 703 studies demonstrating La-induced hormesis, it was found that the maximum biological response to low La concentrations is frequently below 150% of control response, with a geometric mean of 142% at 56 μM, while the geometric mean concentration of the no-observed-adverse-effect-level (NOAEL) was 249 μM^20^. Consequently, one can expect no toxic effects in plants if La is applied between the range of approximately 50 and 250 µM, which will depend on the plant genotype, pH of the growth media, La doses below the NOAEL, and time of exposure to La^13,20^. A summary of the aforementioned findings is presented in Fig. 1.

In preliminary experiments, we tested the effect of a number of different La concentrations on tulip plant growth, development, and nutrient concentration in different plant tissues^17,21^. Furthermore, we analyzed the literature on La dosage triggering beneficial effects in other plant species. For instance, in tomato (*Solanum lycopersicum* L.), the application of 40.78 μM La in the nutrient solution increased yield and reduced Cd accumulation in fruits^22^. In rose (*Rosa hybrida* L.), the application of 50 μM La increased fresh weight and water balance, maintained flower diameter and the stability of membranes in petals, decreased the solute exosmosis and the respiration rate, and prolonged vase life for 2–3 d more than the control^23^. In Easter lily, the application of 30–90 µM LaCl_3_ delayed flower senescence by stimulating the antioxidant defense system and water retaining capacity^15^. Nevertheless, there is scant information available on the use of La^3+^ in postharvest aimed at increasing the duration of the tulip flower stems.

Based on these reports and our experience, we hypothesized that low doses of lanthanum may delay senescence and improve postharvest quality in cut tulip flowers. Concomitantly, we decided to perform further analyses by comparing the effect of applying 0 (control) and 40 μM La on flower quality indicators and senescence inhibition of cut tulip flowers. In our experiment, we evaluated the responses of 15 tulip cultivars to two sources of lanthanum [LaCl_3_ and La(NO_3_)_3_ × 6H_2_O] in order to gain better insight into genotype responses to this beneficial element supplied in the treatment solution.

## Results

Regarding ascorbic acid (AsA), it has been widely used as a key component of preservative solutions for postharvest management of cut flowers. Pulse and continuous treatments with AsA (50–1000 mg/L) have been proved to delay senescence, enhance quality, and prolong the vase life of different cut flowers^5,6,24–29^. We further analyzed the literature on AsA dosage triggering preservative effects on cut flowers (Table 1) and selected 0.2 g/L (i.e. 200 mg/L) to test its effect on tulips.

**Table 1:** Concentrations of ascorbic acid (AsA) that have been evaluated in preservative or holding/vase solutions in some cut flowers.

Species	AsA concentration tested (mg/L)	Treatment application	Reference
Tuberose [*Polianthes tuberosa* L.]	50, 100, 150 and 200	Continuous	Anjum et al*.* (2001)^24^
Sunflower (*Helianthus annuus* L.)	150	Pulse	Mensuali-Sodi & Ferrante (2005)^25^
Rose [*Rosa hybrida* L. cv. Samantha]	1056.72	Pulse	Jin et al*.* (2006)^26^
Red ginger [*Alpinia purpuratta* (Vieill.) K. Schum]	100, 500 and 1000	Pulse	Ieamtim et al*.* (2008)^27^
Lisianthus [*Eustoma grandiflorum* (Raf.) Shinners]	50, 100, 200	Continuous	Azizi et al*.* (2015)^6^
Rose (*Rosa hybrida* L. cv. First Red)	150	Continuous	Bhaskar et al*.* (2017)^28^
Chrysanthemum (*Dendranthema gradiflora* [Ramat.] Kitam.)	100, 200 and 300	Continuous	Budiarto (2019)^29^

### Bud length and diameter

**Figure 2:**
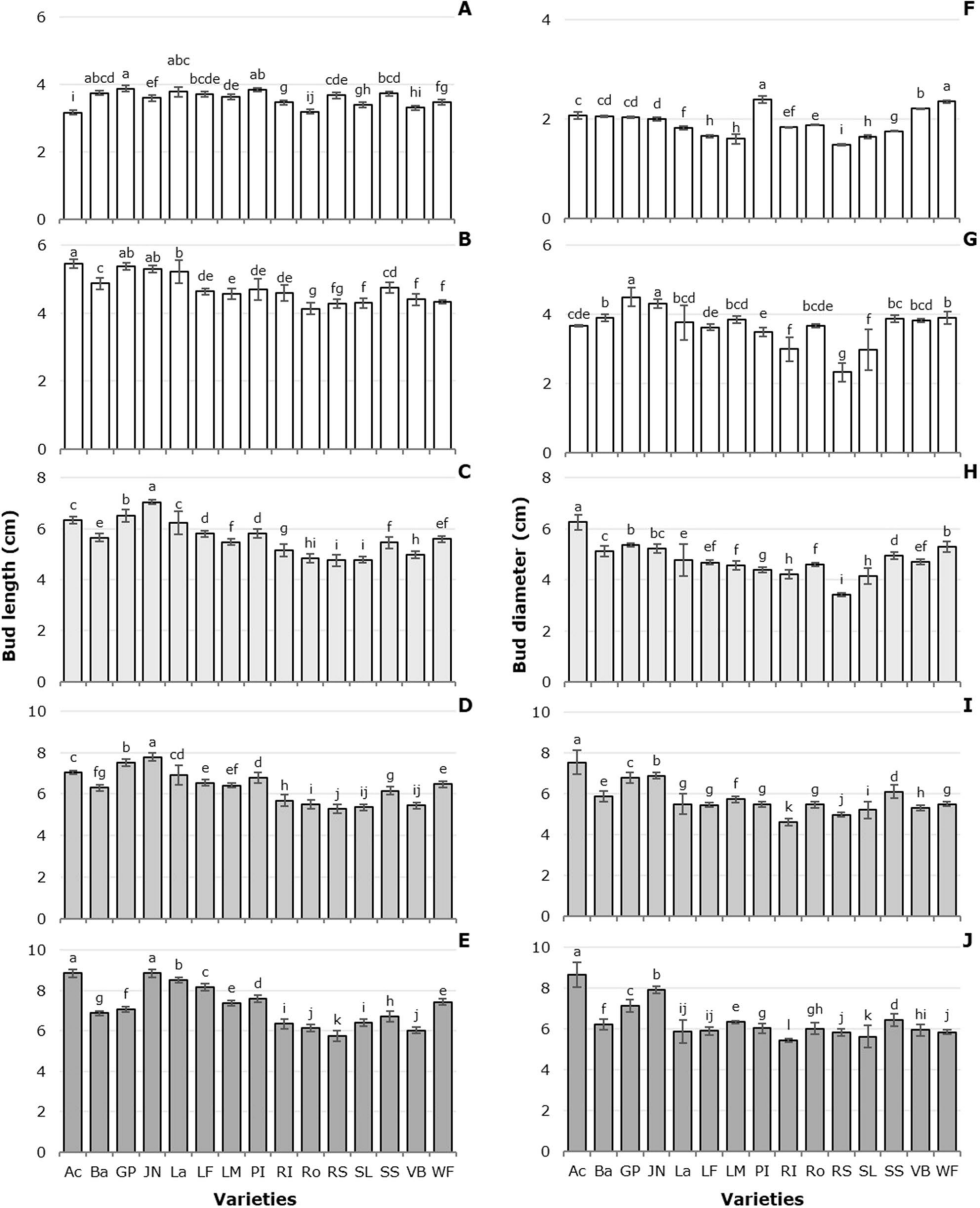
Tulip bud length and diameter in postharvest as a function of the cultivars tested. (**A)** and (**F)**: 3 dac; (**B)** and (**G)**: 5 dac; (**C)** and (**H)**: 7 dac; (**D)** and (**I)**: 9 dac; (**E)** and (**J)**: 11 dac. Different letters in each subfigure and assessment date indicate statistical differences according to the LSD test with *P* ≤ 0.05. (Bud length: 3 dac *P* =  < 0.0001; 5 dac *P* =  < 0.0001; 7 dac *P* =  < 0.0001; 9 dac *P* =  < 0.0001; 11 dac *P* =  < 0.0001. Bud diameter: 3 dac *P* =  < 0.0001 = ; 5 dac *P* =  < 0.0001; 7 dac *P* =  < 0.0001; 9 dac *P* =  < 0.0001; 11 dac *P* =  < 0.0001). Ac: Acropolis, Ba: Barcelona, GP: Golden Parade, JN: Jan van Nes, La: Lalibela, LF: Laura Fygi, LM: Lefeber´s Memory, PI: Pink Impression, RI: Red Impression, RS: Red Shine, Ro: Rosario, SL: Snow Lady, SS: Synaeda Show, VB: Violet Beauty, WF: World´s Favorite. dac: days after cutting. Data are means ± SD of six biological replicates.

Due to the cultivar factor, bud length and diameter increased from 3 to 11 days after cutting (dac; Fig. 2A–J), with the highest elongation rates occurring from 3 to 5 dac (Fig. 2A,B,F,G). The cultivars Acropolis and Jan van Nes had the greatest bud length (Fig. 2E). The bud diameter in all cultivars increased over time (Fig. 2F–J), displaying its highest values at 11 dac (Fig. 2J). The highest bud diameter values at 11 dac were recorded in Acropolis, followed by Jan van Nes and Golden Parade (Fig. 2J).

**Figure 3:**
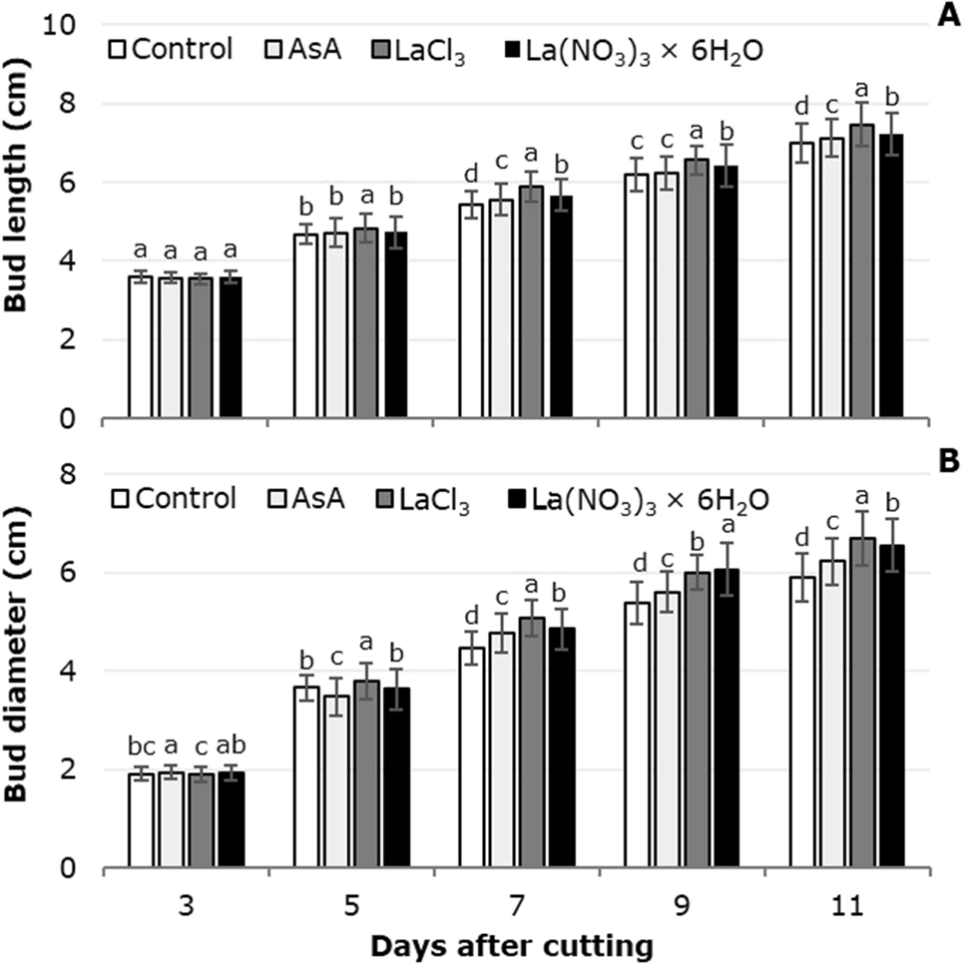
Tulip bud length (**A**) and diameter (**B**) in postharvest as a function of the treatment solutions used. Different letters in each subfigure and assessment date indicate statistical differences according to the LSD test with *P* ≤ 0.05. (Bud length: 3 dac *P* = 0.0016; 5 dac *P* =  < 0.0001; 7 dac *P* =  < 0.0001; 9 dac *P* =  < 0.0001; 11 dac *P* =  < 0.0001. Bud diameter: 3 dac *P* =  < 0.0001; 5 dac *P* =  < 0.0001; 7 dac *P* =  < 0.0001; 9 dac *P* =  < 0.0001; 11 dac *P* =  < 0.0001). Control: distilled water; AsA: L-ascorbic acid, 0.2 g/L; LaCl_3_: lanthanum(III) chloride, 40 µM; La(NO_3_)_3_ × 6H_2_O: lanthanum(III) nitrate hexahydrate, 40 µM. Data are means ± SD of six biological replicates.

Figure 3A shows the bud length as affected by the treatment solution. From 5 dac significant differences among treatments were observed. With LaCl_3_ in the solution, flower stems showed the greatest bud length in measurements performed 5, 7, 9 and 11 dac. The second highest mean was observed in flower stems treated with La(NO_3_)_3_ at the same time points analyzed. Indeed, at 11 dac the use of LaCl_3_ in the treatment solution produced better results than the other treatments, having a bud length of 7.4 cm, while with ascorbic acid and La(NO_3_)_3_ it was 7.0 and 7.2 cm, respectively. Stems treated with LaCl_3_ and La(NO_3_)_3_ recorded the greatest bud diameter at 9 to 11 dac (6.07 and 6.68 cm, respectively). From 7 dac the control treatment showed the smallest bud diameter. Thus, at 11 dac the means of LaCl_3_-treated flowers exceeded those observed in flowers treated with ascorbic acid and the control by 7.4 and 13.2% (Fig. 3B).

### Stem length and curvature

The Acropolis cultivar showed the greatest stem length in vase. The shortest stem length was found in Rosario. The highest relative stem elongation was observed when the initial stem length was the shortest 3 dac, in the cultivars Laura Fygi and Rosario (Table 2).

**Table 2:** Flower stem length (cm) and relative stem elongation (in parenthesis) in postharvest of evaluated tulip cultivars.

Cultivar	Days after cutting				
	3	5	7	9	11
Acropolis	50.2 ± 0.27 a (100)	52.8 ± 0.54 a (105.4)	55.8 ± 0.84 a (111.2)	58.5 ± 1.16 a (116.6)	60.6 ± 1.11 a (120.8)
Barcelona	35.1 ± 0.16 j (100)	37.2 ± 0.67 i (105.7)	38.6 ± 0.94 j (109.9)	40.3 ± 1.22 l (114.7)	42.1 ± 1.02 j (119.9)
Golden Parade	41.1 ± 0.21 f. (100)	44.6 ± 1.02 e (108.6)	48.6 ± 0.84 f. (118.3)	52.0 ± 0.69 f. (126.5)	56.8 ± 1.84 c (138.2)
Jan van Nes	41.0 ± 0.31 f. (100)	47.7 ± 0.95 c (116.1)	52.2 ± 1.45 c (127.1)	55.0 ± 1.37 c (134.1)	56.8 ± 1.26 c (138.4)
Lalibela	42.0 ± 0.10 e (100)	45.5 ± 0.68 d (108.2)	48.5 ± 1.09 f. (115.5)	51.1 ± 1.40 g (121.7)	54.1 ± 1.57 f. (128.9)
Laura Fygi	31.1 ± 0.14 l (100)	36.2 ± 0.25 j (116.3)	40.9 ± 0.69 h (131.6)	45.0 ± 0.93 j (144.6)	49.8 ± 1.31 h (160.2)
Lefeber´s Memory	40.1 ± 0.12 g (100)	43.1 ± 0.40 f. (107.6)	46.0 ± 0.54 g (114.8)	49.1 ± 1.04 i (122.4)	51.9 ± 1.42 g (129.4)
Pink Impression	38.1 ± 0.09 i (100)	41.9 ± 0.88 g (110.0)	46.4 ± 1.87 g (121.8)	49.8 ± 2.32 h (130.9)	55.8 ± 1.54 d (146.5)
Red Impression	46.1 ± 0.07 c (100)	48.1 ± 0.41 c (104.2)	50.2 ± 0.72 e (108.9)	52.6 ± 0.91 e (114.2)	54.9 ± 1.45 e (119.1)
Red Shine	25.1 ± 0.09 m (100)	28.6 ± 0.26 k (113.8)	31.7 ± 0.29 k (126.2)	35.1 ± 0.60 m (139.8)	38.2 ± 0.82 l (152.1)
Rosario	39.0 ± 0.16 h (100)	39.6 ± 0.48 h (101.7)	40.0 ± 0.57 i (102.8)	40.5 ± 0.66 l (104.1)	41.6 ± 0.90 k (106.8)
Snow Lady	44.1 ± 0.17 d (100)	47.9 ± 1.44 c (108.7)	51.7 ± 1.67 d (117.3)	54.0 ± 1.80 d (122.6)	55.4 ± 0.61 d (125.7)
Synaeda Show	46.0 ± 0.14 c (100)	49.9 ± 0.98 b (108.4)	53.1 ± 1.30 b (115.4)	56.1 ± 1.59 b (121.8)	59.6 ± 2.00 b (129.5)
Violet Beauty	33.0 ± 0.20 k (100)	35.9 ± 0.66 j (108.7)	38.6 ± 1.10 j (116.8)	41.7 ± 1.52 k (126.3)	44.3 ± 2.20 i (134.3)
World´s Favorite	46.5 ± 0.31 b (100)	49.9 ± 0.53 b (107.3)	52.6 ± 0.67 c (113.1)	55.0 ± 0.95 c (118.3)	57.1 ± 1.04 c (122.8)

Flower stem length was estimated from the junction of the stem and bulb to the start of the receptacle, while the relative stem elongation was calculated with respect to the measurement 3 dac (100%), as described in “Materials and methods”. Different letters in the same column indicate statistical differences among cultivars according to the LSD test with *P* ≤ 0.05. (3 dac *P* =  < 0.0001; 5 dac *P* =  < 0.0001; 7 dac *P* =  < 0.0001; 9 dac *P* =  < 0.0001; 11 dac *P* =  < 0.0001). In the case of stem length, data are means ± SD of six biological replicates.

**Figure 4:**
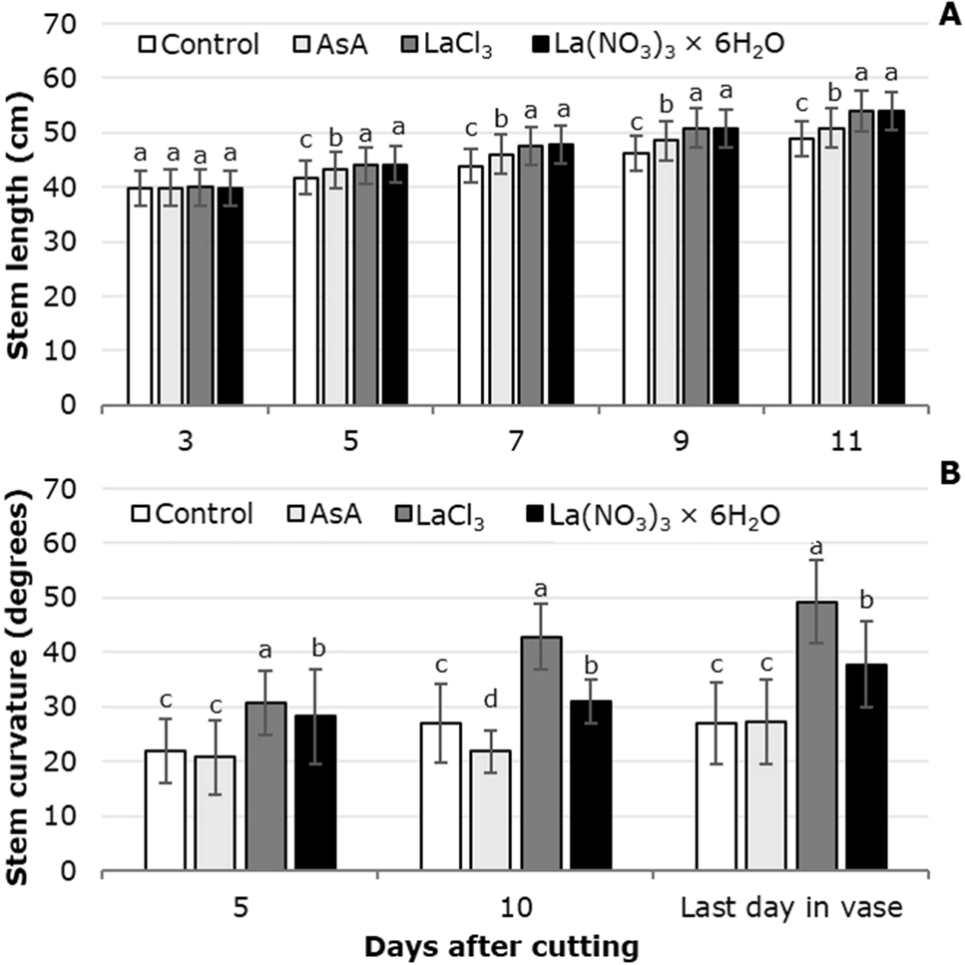
Tulip stem length (**A**) and curvature (**B**) in postharvest as a function of the treatment solutions used. Different letters in each subfigure and assessment date indicate statistical differences according to the LSD test with *P* ≤ 0.05. (Stem length: 3 dac *P* = 0.2847; 5 dac *P* =  < 0.0001; 7 dac *P* =  < 0.0001; 9 dac *P* =  < 0.0001; 11 dac *P* =  < 0.0001. Stem curvature: 5 dac *P* =  < 0.0001; 10 dac *P* =  < 0.0001; last day in vase *P* =  < 0.0001). Control: distilled water; AsA: L-ascorbic acid, 0.2 g/L; LaCl_3_: lanthanum(III) chloride, 40 µM; La(NO_3_)_3_ × 6H_2_O: lanthanum(III) nitrate hexahydrate, 40 µM. Data are means ± SD of six biological replicates.

Stem length increased during vase life, regardless of the treatment solution tested. At 5, 7, 9 and 11 dac the greatest stem length was observed with La(NO_3_)_3_, surpassing the control by 5.4, 8.1, 9.2 and 10.3% and the solution with ascorbic acid by 2.0, 3.5, 4.5 and 5.7%, respectively (Fig. 4A). The relative stem elongations observed were 21.3, 27.4, 35.2 and 35.5% in the control, AsA, LaCl_3_ and La(NO_3_)_3_ × 6H_2_O, respectively. Stem curvature in postharvest showed significant changes as a result of the treatment solutions used. On the three evaluation dates (i.e. 5 and 10 dac, and on the last day in the flower vase) the greatest stem curvature was observed in the treatment solution with LaCl_3_, followed by the treatment solution with La(NO_3_)_3_ × 6H_2_O. By contrast, the smallest curvature angle was recorded in the control (Fig. 4B).

**Figure 5:**
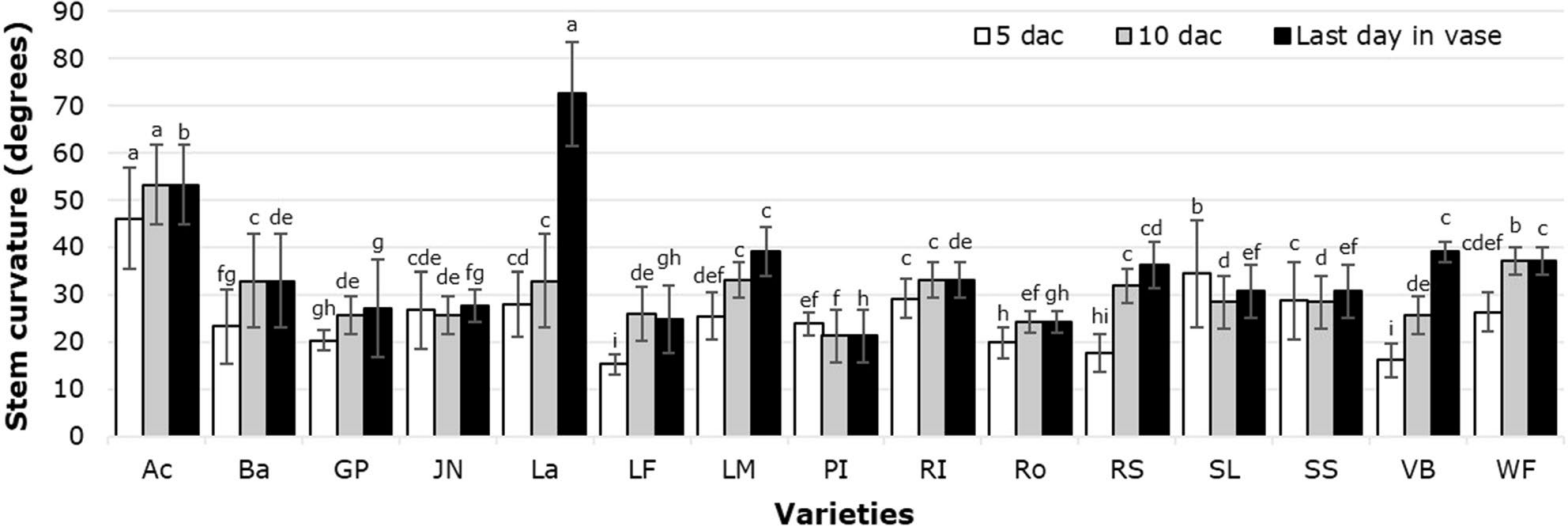
Tulip stem curvature in postharvest as a function of the cultivars tested. Different letters in each subfigure and assessment date indicate statistical differences according to the LSD test with *P* ≤ 0.05. (5 dac *P* =  < 0.0001; 10 dac *P* =  < 0.0001; last day in vase *P* =  < 0.0001). Ac: Acropolis, Ba: Barcelona, GP: Golden Parade, JN: Jan van Nes, La: Lalibela, LF: Laura Fygi, LM: Lefeber’s Memory, PI: Pink Impression, RI: Red Impression, RS: Red Shine, Ro: Rosario, SL: Snow Lady, SS: Synaeda Show, VB: Violet Beauty, WF: World´s Favorite. dac: days after cutting. Data are means ± SD of six biological replicates.

A wide variation in stem curvature among cultivars in vase was observed, with statistically significant differences occurring at 5 and 10 dac, and on the last day in vase. During the last measurement, the cultivars Lalibela and Acropolis displayed the greatest curvature (Fig. 5).

**Figure 6:**
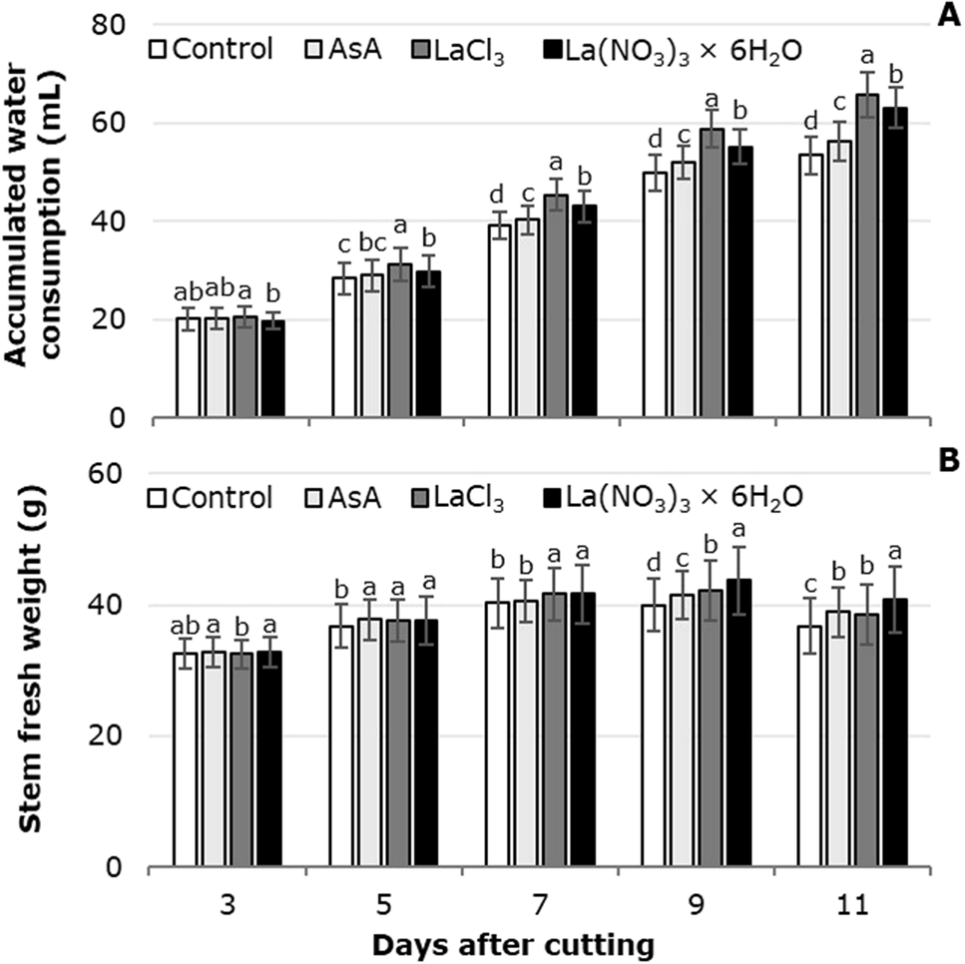
Accumulated solution uptake (**A**) and fresh weight of tulip stems (**B**) in postharvest as a function of the treatment solutions used. Different letters in each subfigure and assessment date indicate statistical differences according to the LSD test with *P* ≤ 0.05. (Accumulated solution uptake: 3 dac *P* = 0.0687; 5 dac *P* =  < 0.0001; 7 dac *P* =  < 0.0001; 9 dac *P* =  < 0.0001; 11 dac *P* =  < 0.0001. Fresh weight of tulip stems: 3 dac *P* = 0.0027; 5 dac *P* = 0.0275; 7 dac *P* = 0.0002; 9 dac *P* =  < 0.0001; 11 dac *P* =  < 0.0001). Control: distilled water; AsA: L-ascorbic acid, 0.2 g/L; LaCl_3_: lanthanum(III) chloride, 40 µM; La(NO_3_)_3_ × 6H_2_O: lanthanum(III) nitrate hexahydrate, 40 µM. Data are means ± SD of six biological replicates.

The application of ascorbic acid, LaCl_3_ and La(NO_3_)_3_ increased solution uptake in the tulip flower stems. Particularly with LaCl_3_ the highest values were observed at 3, 5, 7, 9 and 11 dac, surpassing the control by 5, 11, 15, 18 and 24%, respectively, which showed the lowest values. The mean solution uptake per gram of stem fresh biomass weight (FBW) was 1.44, 1.44, 1.71 and 1.54 mL in the control, AsA, LaCl_3_ and La(NO_3_)_3_ × 6H_2_O, respectively (Fig. 6A).

At 5 and 7 dac, the greatest stem weight was found by adding La^3+^, either in the form of chloride or nitrate. The highest fresh weight (43.7 g) was found 9 dac in stems treated with La(NO_3_)_3_, followed by those treated with LaCl_3_ (42.1 g) and ascorbic acid (41.5 g); the control had the lowest weight (Fig. 6B).

**Figure 7:**
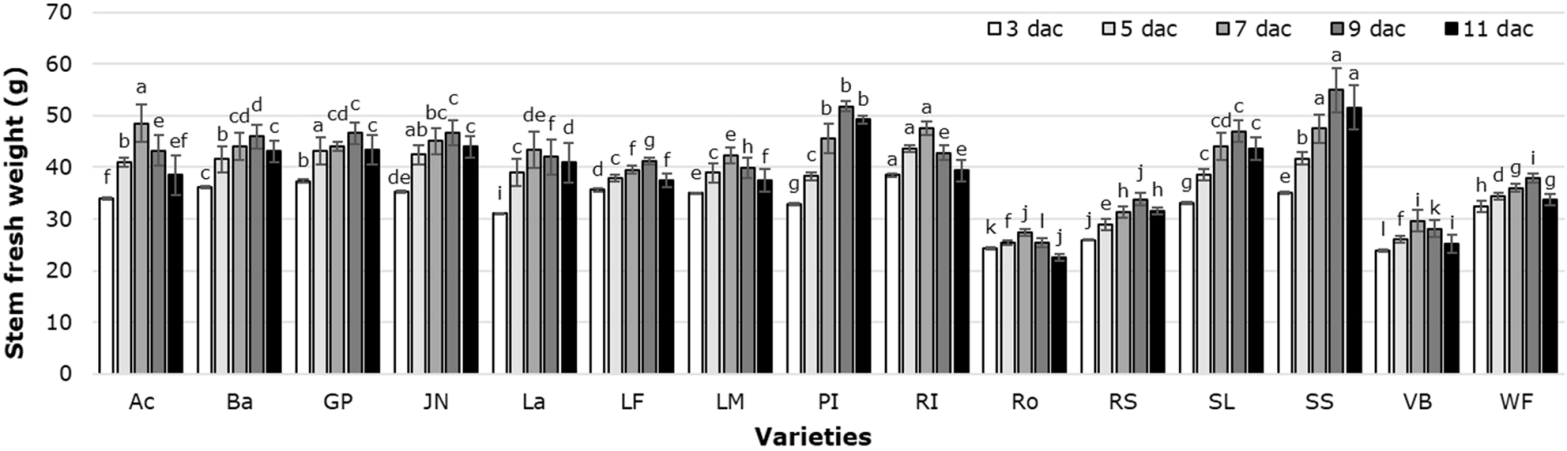
Fresh weight of tulip stems in postharvest as a function of the cultivars tested. Different letters in each assessment date indicate statistical differences according to the LSD test with *P* ≤ 0.05. (3 dac *P* =  < 0.0001; 5 dac *P* =  < 0.0001; 7 dac *P* =  < 0.0001; 9 dac *P* =  < 0.0001; 11 dac *P* =  < 0.0001). Ac: Acropolis, Ba: Barcelona, GP: Golden Parade, JN: Jan van Nes, La: Lalibela, LF: Laura Fygi, LM: Lefeber’s Memory, PI: Pink Impression, RI: Red Impression, RS: Red Shine, Ro: Rosario, SL: Snow Lady, SS: Synaeda Show, VB: Violet Beauty, WF: World’s Favorite. dac: days after cutting. Data are means ± SD of six biological replicates.

From 3 dac, stem fresh weight increased, reaching its peak values in most cultivars between 7 and 9 dac. With the results obtained, we could identify cultivars that maintain their constant weight during postharvest, including World´s Favorite, Violet Beauty, Laura Fygi and Rosario, in which the difference between the weight at 3 dac and the maximum weight is less than 6 g (Fig. 7).

## Discussion

Lanthanum’s major uses include petroleum cracking and chemical industry catalysts. Furthermore, La can be used in glasses, studio lights and projectors, lighters and torches, electron cathodes, and scintillators, among others. Lanthanum carbonate is used as a phosphate binder in cases of renal failure or hypophosphatemia in humans. Additionally, La may have positive effects on plant physiology and may improve some crop yield indicators^30^. It has been less frequently studied in ornamental plants or cut flowers than it has in cereal grains and industrial crops^8^.

In cut tulip flowers, bud length of different commercial cultivars may range from 4.8 to 9.2 cm^31^. Under our experimental conditions, these values varied from 3.2 to 3.9 cm 3 dac, from 4.1 to 5.5 cm 5 dac, from 4.8 to 7.1 cm 7 dac, and from 5.3 to 7.8 cm 9 dac, respectively. In general, values observed at the end of vase life (11 dac) in our study are in full agreement with those reported elsewhere^31^. On average, the bud diameter in the cultivars evaluated 3, 5, 7, 9 and 11 dac was 1.9, 3.6, 4.8, 5.8 and 6.3 cm, respectively. In a previous study, we reported that the longest vase life (13 days) occurred in Laura Fygi treated with 40 µM La (either as LaCl_3_ or as La[NO_3_]_3_), which was associated with greater water uptake and concentrations of sugars, proteins and chlorophylls^9^. In most plant species, flowering is a highly intricate process that depends on both endogenous and exogenous factors. Regarding endogenous factors, flowering may be determined by local elongation growth and local ion accumulation, which in turn depend on the genetic background of the genotype studied. External or exogenous factors include temperature, quality and quantity of light, and duration of both light and darkness^32^. Senescence is also a highly regulated active process, which involves metabolic changes that are part of a genetically-based program leading to death of the cells involved^33^. Flower senescence refers to a series of deterioration phases involving petals, corolla and tepals, and translocation of nutrients out of the flower to sustain growth and development of seeds^34,35^. Under our experimental conditions, La improved flower quality parameters and hence delayed senescence.

Stem length was different among the cultivars evaluated. In a study involving the tulip cultivars Apeldoorn, Paul Richter and Rose Copland, stem elongation was associated with a higher concentration and activity of gibberellins, while differences in stem length were observed among cultivars^36^. In addition, a higher concentration of gibberellins increased transport of auxins and enhanced their biosynthesis^37^.

Regarding the treatment solutions employed, from 5 dac the LaCl_3_ treatment stood out by producing the highest bud length and bud diameter values. The AsA treatment generally resulted in higher bud length and diameter values than the control treatment. It has been well documented that in cut flowers, AsA promotes stomatal closure, decreasing respiration and water loss by transpiration^38^.

We observed a positive relationship between bud length and stem length measured during vase life, as a function of the treatment solution. In particular, stem length was always greater when La was added, regardless of the source used. In Ile de France tulip, the use of 10 µM La in the nutrient solution during the production cycle increased bud length and diameter compared to the control^17^. The effects of La on cell division and elongation processes have not yet been fully elucidated and they have been generally studied during the production cycle, by supplying La to the soil, in nutrient solutions or as foliar sprays, with differential results among species. In durum wheat (*Triticum durum* L.), there was a reduction in root length and a decrease in the mitotic index, from 21.53% in the control to 9.91, 1.90 and 0.69% in plants treated with 0.01, 1.0 and 10.0 mM La^3+^, respectively^12^. In tulip, the application of 2.5 mM La^3+^ inhibited stem elongation, while the addition of 25 mM La^3+^ resulted in smaller flower diameters compared to the control^16^.

In daylily (*Hemerocallis lilioasphodelus* L.), narcissus (*Narcissus* spp. L.) and snapdragon (*Antirrhinum majus* L.), the application of La^3+^ has been shown to favor different yield attributes and floral stem growth^39,40^. In soybean (*Glycine max* L.), La induced cell division in roots and consequently increased the mitotic index, but the occurrence of abnormalities such as c-metaphases, which occur simultaneously in the root, adversely affect their growth^41^.

Stem curvature (negative gravitropism) is another valuable attribute to be evaluated in cut flowers, being a problem that affects the quality of the stems on the market and thus lowers their sales potential. In order to reduce negative gravitropism in tulip floral stems it is necessary to select cultivars that show minimum stem growth in postharvest, as stem elongation is a genetically controlled trait passed down by the parents to their offspring^42^. In our study, differences among the cultivars evaluated are evident. Importantly, Labilela and Acropolis showed the most pronounced curvatures at the end of vase life (> 50°). Crossing of genotypes that show minimum stem elongation in postharvest can generate new cultivars with less stem growth, and therefore less curvature.

It has been demonstrated that cytosolic Ca^2+^ acts as a second messenger involved in the gravitropic curvature of floral stems, since it causes a redistribution of auxins along the stems and increases the production of ethylene, thereby generating the curvature^43^. La^3+^ inhibits several Ca-dependent processes, blocks Ca channels and stimulates Ca^2+^-ATPase activity, preventing an increase in cytosolic Ca^2+^ concentration^44^, thus reducing stem curvature. However, herein we observed increased stem curvature caused by the La treatment, which is positively associated with stem elongation in the vase. This means that with greater stem elongation the curvature thereof increases in the vase. In snapdragon stems, LaCl_3_ inhibited stem curvature in a horizontal and vertical position, since the La^3+^ decreased stem elongation and inhibited various processes dependent on gravitropism^43^. Furthermore, high doses of LaCl_3_ (20 to 30 mM) decreased the stem elongation rate^40^, which indicates that LaCl_3_ supply inhibits stem curvature as a result of its antagonism against Ca^39^.

In tulip cultivars Peer Gynt, Maureen and Kingsblood, the application of 25 mM LaCl_3_ resulted in stem curvature and elongation similar to that observed in snapdragon stems. It is well documented that stem responses to gravistimulation are differential among cultivars, probably caused by a genetic variation in stem elongation, which is positively correlated with stem curvature in response to LaCl_3_^16^.

During the final stage of senescence, one of the most evident symptoms is the loss of fresh weight due to dehydration, mainly of the petals, which causes wilting^45^. In cut flowers, vase life is determined by curvature of the flower stem, dehydration and loss of fresh weight^46^. In the cultivars evaluated, we observed a positive relationship between the accumulated water and the weight of the stems in the vase; this trend was also observed in response to the treatment solutions evaluated. Indeed, we have previously demonstrated that La treatments (either as LaCl_3_ or as La[NO_3_]_3_) extend the vase life of tulip flowers from 9 days (in the control) to 12 days^9^. In the cultivars Burgundy, Gander, Don Quichott, Upstar and King Blood, vase life had a maximum of 9.0 days, with a minimum of 5.8 days^47^, while the cultivar Triumph registered 11.8 vase days when treated with a solution containing 75 mL/L humic acid + 10 g/L NPK, while the control (distilled water) resulted in 6.3 vase days^48^. This behavior was associated with the genetic background of each cultivar evaluated, the culture management and the environment.

In regard to the treatment solutions tested, the longer vase life observed with La^3+^ was due to the greater stem length, stem fresh weight and solution uptake. As mentioned before, La^3+^ may exert an antagonistic action with ethylene and increase cellular antioxidant activity, reducing the formation of reactive oxygen species (ROS), which retards senescence^49^.

It is well known that La promotes antioxidant activity in plants. For instance, the application of 40 µM LaCl_3_ in rice stimulated the redox system, but concentrations higher than 40 µM drastically depress such activity^50^. In cut Easter lily flowers, La significantly increased the activities of antioxidant enzymes, while decreasing the concentrations of reactive oxygen species, as compared to the control^11^. Similar responses have been observed in tomato^51,52^ and rice^49,53^. In our study, we tested the effect of LaCl_3_ and La(NO_3_)_3_ × 6H_2_O at a concentration of 40 µM each on floral quality indicators of 15 commercial tulip cultivars. We measured some physiological and biochemical responses of tulips, though the effect of La on the antioxidant capacity of cut flowers was beyond the scope of our study. In future approaches, we are planning to perform further biochemical and molecular analyses aimed at deciphering the effect of different sources of La on the antioxidant capacity of different plant genotypes.

Rare Earth Elements such as La can accumulate in the environment with potential effects in living organisms and ecosystems. Hence, special attention should be paid to control for desired effects, such as stimulation of productivity, while maintaining quality at acceptable standards^54^. Though La has not yet demonstrated a defined biological role in humans, it can indeed trigger proliferation, osteogenic differentiation, and mineralization of MC3T3-E1 cells^55^, while its role as an effective phosphate binder that can control serum phosphate in patients with hyperphosphatemia is well recognized^56^. Nevertheless, surpassing the toxicological threshold for adverse effects may lead to disruption of diverse food chains, with potential implications for the homeostasis of living systems^57^. Consequently, according to current scientific surveys, environmental standards should maintain La concentrations below 250 µM. Currently, the development of novel analytical technologies may expand the application of La and other REE in parallel^58^.

Discoveries on the effects of La on cut tulip flower senescence and quality parameters are of paramount importance to determine the role of this beneficial element on postharvest management of ornamental species. Herein we have demonstrated that La delays senescence and improves postharvest quality in cut tulip flowers. Particularly, La increased bud length and diameter, and stem length, and consequently stem curvature was also increased. Flower stems treated with LaCl_3_ showed the highest solution uptake. Furthermore, relative stem elongation was the highest in flower stems exposed to either LaCl_3_ or La(NO_3_)_3_, while La(NO_3_)_3_ increased stem fresh weight. Regarding the cultivars, Laura Fygi and Rosario registered the highest relative stem elongation, while Lalibela and Acropolis displayed the greatest stem curvature on the last day in vase.

Since lanthanum-containing compounds are commercialized by different companies worldwide, the use of this beneficial element in holding solutions for cut flower postharvest management is feasible. Importantly, the cost of each gram of LaCl_3_ ranges between 0.002 and 0.7 US dollar (USD) cents, while the price of each gram of La(NO_3_)_3_ × 6H_2_O is around 0.2 and 0.4 USD cents. Therefore, the cost of 100 mL of the solution containing 40 µM La (needed for each tulip floral stem) is approximately 0.15–0.11 USD cents, depending on the use of LaCl_3_ or La(NO_3_)_3_ × 6H_2_O, respectively. If agrochemical companies are able to develop commercial solutions containing La at a lower price, then the use of this element for cut flowers can be expanded.

## Materials and methods

### Treatment and experimental design

To test the effect of lanthanum on cut tulip flowers, we used the stems of 15 commercial tulip cultivars. Tulip bulbs of 12 + grade were bought from the Mexican company Akiko, which is the exclusive distributor of the Dutch company Jan de Wit en Zonen B. V. (https://www.jandewitenzonen.com/en/home/) in Mexico. It is important to note that the number 12 refers to the circumference length in cm, while the + symbol is used in commercialization to indicate bulbs which are 12 cm or more in size. Bulbs were sown individually in 2.25 L pots, which contained a 30:70 (v:v) mixture of peatmoss and a volcanic gravel (particle size 3 mm), respectively. Plants were irrigated with half-strength Steiner nutrient solution^59^, which was prepared using analytical grade reagents (J. T. Baker; Phillipsburg, NJ, USA), and the pH was adjusted to 5.5 every other day. This initial phase of the experiment was carried out under greenhouse conditions with a minimum temperature of 1.5 °C and a maximum of 22 °C, light intensity of 165.4 µmol/m/s (produced with a 50% shade mesh), and average relative humidity of 84%. A total of 150 mL of the Steiner nutrient solution were applied to each individual pot every other day. Plants reached the mature stage at different time points, which depended on each cultivar evaluated. In general, flower stems were cut when flower buds reached < 50 of their color, which corresponded to stage 5 of the scale developed elsewhere^60^. Immediately after cut, flower stems were placed in individual vases containing the solutions to be tested, and then evaluated in the laboratory.

In the treatment solution of cut tulip flowers, two sources of lanthanum at 40 µM La each were tested: lanthanum chloride (LaCl_3_) and lanthanum nitrate hexahydrate [La(NO_3_)_3_ × 6H_2_O], while L-ascorbic acid (AsA) at 0.2 g/L was used as a reference solution. The absolute control was distilled water. In all cases, distilled water was used to prepare the treatment solutions tested. L-ascorbic acid (AsA), LaCl_3_ and La(NO_3_)_3_ × 6H_2_O were provided by Sigma Aldrich (Darmstadt, Germany). One assay per cultivar was established in order to test our treatments, so that 15 independent trials were carried out in the laboratory at room temperature in a 15 × 4 factorial experiment with completely randomized distribution. The study factors were the commercial tulip cultivar (Ac: Acropolis, Ba: Barcelona, GP: Golden Parade, JN: Jan van Nes, La: Lalibela, LF: Laura Fygi, LM: Lefeber’s Memory, PI: Pink Impression, RI: Red Impression, RS: Red Shine, Ro: Rosario, SL: Snow Lady, SS: Synaeda Show, VB: Violet Beauty, and WF: World’s Favorite) and the treatment solution (distilled water as absolute control; AsA, 0.2 g/L as reference solution; LaCl_3_, 40 µM; and La(NO_3_)_3_ × 6H_2_O, 40 µM). Our experimental unit was a 500 mL glass jar containing one of the four treatment solutions and two flower stems of each cultivar. Consequently, we evaluated 180 experimental units in total. The environmental conditions in the laboratory during the conducting of the experiment were as follows: mean day/night temperature of 20 °C/17 °C, respectively; mean relative humidity of 40%; and 12 h light (12 µmol/m/s light intensity) photoperiod. The stems of all cultivars received the same agronomic and nutritional management during the experimental period. Stems were cut at the beginning of flowering and exposed to the treatment solutions as previously described^61^. All treatment solutions tested [control, L-ascorbic acid, LaCl_3_ or La(NO_3_)_3_ × 6H_2_O] were applied as steady solutions throughout the duration of the experiment.

### Variables evaluated

Once flower stems were cut (day 0), they were immediately placed in glass jars containing the different treatment solutions to be tested. Then, at 3, 5, 7, 9 and 11 days after cutting (dac), we measured bud length and diameter, stem length and curvature, solution uptake and fresh weight of flower stems. Bud length (cm) was measured with a millimeter ruler, from the top of the bud to its receptacle, while flower stem length (cm) was recorded from the junction of the stem and bulb (bottom) to the start of the receptacle (top). Relative Stem Elongation (RSE) was calculated considering the methodology described elsewhere^62^, according to the following equation: RSE = [Stem length in each treatment or variety 3 dac/Stem length in each treatment or variety 11 dac] × 100, with treatment referring to either AsA, LaCl_3_ or La(NO_3_)_3_ × 6H_2_O applied in the solution. Bud diameter (cm) was determined using a digital vernier caliper, according to the methodology described elsewhere^63–65^.

Stem curvature was measured using a 180° protractor at 5 dac, 10 dac and on the last day of vase life and expressed with respect to the angle at time of cutting (0°) as described elsewhere^59^. Solution uptake (mL) was determined by the difference between solution supplied and solution taken up in covered vases, during the different time points measured, using 250 mL graduated cylinders. Fresh weight of flower stems (g) was measured using a digital weighing balance.

### Statistical analysis

Data obtained were subjected to an analysis of variance and the Least Significant Difference (LSD) test with a 95% confidence level, using the Statistical Analysis System software^66^.
